# “All that glitters is not gold”

**DOI:** 10.1002/joa3.12645

**Published:** 2021-10-11

**Authors:** Krishna Kumar Mohanan Nair, Narayanan Namboodiri, Ajitkumar Valaparambil

**Affiliations:** ^1^ Department of Cardiology Sree Chitra Tirunal Institute for Medical Sciences and Technology Thiruvananthapuram India

**Keywords:** atrial flutter, pre‐excited tachycardia, ventricular tachycardia, wide complex tachycardia, WPW syndrome

## Abstract

A 63‐year‐old gentleman presented to the emergency room (ER) with complaints of sudden onset of palpitation for the last 1 hour. He denied any presyncope or syncope. He was tachycardic with pulse rate of 120 beats per minute. His blood pressure was 110/70 mm Hg. A 12‐lead electrocardiogram (ECG) was taken. What is the likely diagnosis?

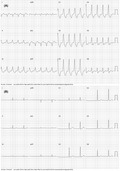

A 63‐year‐old gentleman presented to the emergency room (ER) with complaints of sudden onset of palpitation for the last 1 hour. He denied any presyncope or syncope. He was tachycardic with pulse rate of 120 beats per minute. His blood pressure was 110/70 mm Hg. A 12‐lead electrocardiogram (ECG) was taken which is shown in Figure 1A. What is the likely diagnosis?

**FIGURE 1:**
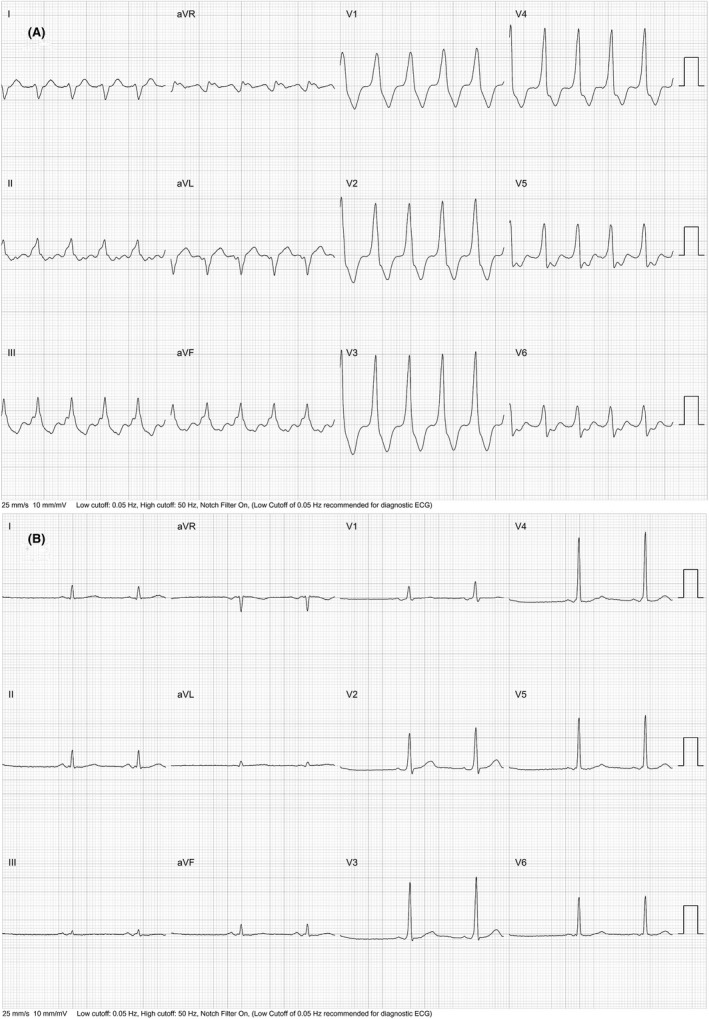
A, showing 12 lead ECG during RBBB tachycardia. B, showing 12 lead ECG during sinus rhythm

The 12 lead ECG shows a regular wide complex tachycardia of atypical right bundle branch block (RBBB) morphology at a rate of 120 beats per minute with right axis deviation (RAD). The QRS complexes showed slurring in the initial upstroke. The differential diagnosis entertained are (a) ventricular tachycardia [VT], (b) supraventricular tachycardia [SVT] with rate‐related aberrancy or preexisting bundle branch block, and (c) pre‐excited SVT.

As per the Brugada algorithm, the tachycardia has R to S nadir interval of more than 100 ms [with RS complex in precordial leads] and monophasic R wave in lead V1 which suggest that the wide complex tachycardia is VT. As per the aVR algorithm the finding of Q wave duration of more than 40 ms in lead aVR is consistent with VT. The R‐wave peak time in lead II is more than 50 ms, which is also diagnostic of VT. Therefore, as per the above electrocardiographic observations, this wide complex tachycardia with atypical RBBB morphology is VT. But it is to be remembered that pre‐excited tachycardia was not included while validating most of VT—SVT discriminating ECG algorithms. If we apply the algorithm that distinguishes between VT and pre‐excited tachycardia in this index case, as follows—predominantly positive QRS complex in leads V4 through V6, no QR complexes in precordial leads, then the wide complex tachycardia will be diagnosed as pre‐excited tachycardia rather than VT.

The patient was electrically cardioverted to sinus rhythm. The 12 lead ECG during sinus rhythm has short PR interval with delta wave consistent with pre‐excitation. The delta wave polarity is positive in precordial leads suggestive of left lateral bypass tract (Figure 1B). In the background of underlying pre‐excitation, the wide QRS tachycardia is diagnosed as pre‐excited tachycardia.

The patient underwent an electrophysiology study which revealed the presence of an antegradely only conducting left lateral bypass tract. A wide complex tachycardia identical to clinical tachycardia was induced in the EP lab. It happened to be atrial flutter with 2:1 A‐V conduction over the left lateral bypass tract (Figure 2). The left lateral bypass tract was located at 3′O clock of the mitral annulus (Figure 3A) and successfully ablated with the delivery of radiofrequency energy (Figure 3B). The atrial flutter persisted and it was proved to be cavotricuspid isthmus (CTI) dependent by entrainment [concealed entrainment with 2 × post pacing interval (PPI) − 2 × tachycardia cycle length (TCL) less than 30 ms] (Figure 4A). Radiofrequency ablation at the CTI region terminated the flutter (Figure 4B) and bidirectional block across the CTI region was ensured.

The wide complex tachycardia, apparently looking as VT, happened to be a case of WPW syndrome with pre‐excited CTI dependent atrial flutter having 2:1 A‐V conduction across the antegradely only conducting left lateral bypass tract.

**FIGURE 2:**
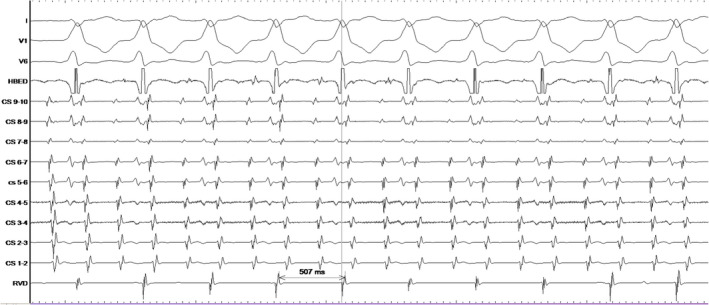
Showing selected surface ECG and intracardiac EGMs during the tachycardia. It shows pre‐excited atrial flutter with 2:1 AV conduction

**FIGURE 3:**
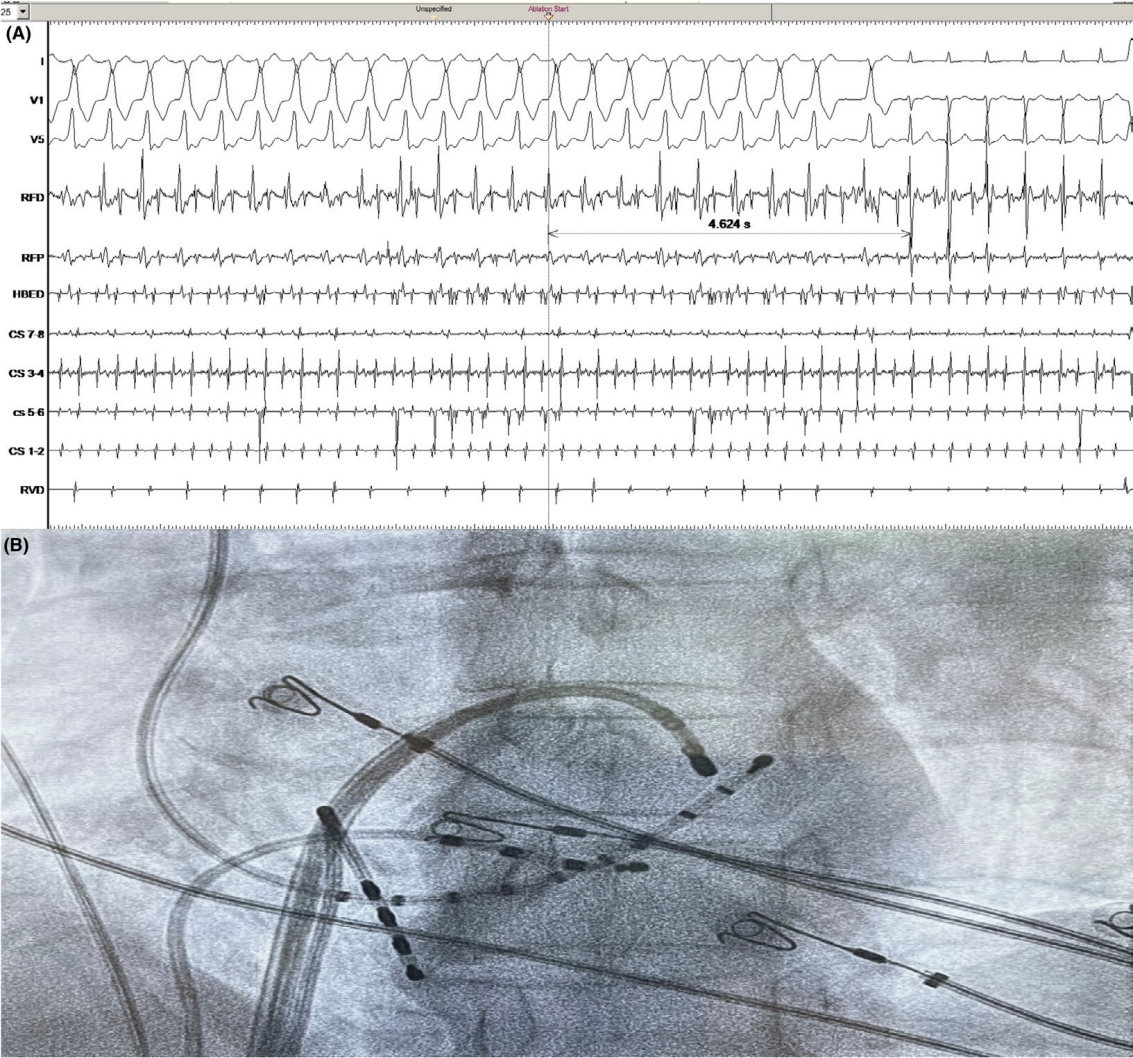
A, represents surface ECG and intracardiac EGMs during the RF ablation. It shows that on delivery of RF energy there is loss of pre‐excitation with the continuation of atrial flutter. B, represents fluoroscopic image during RFA. Mapping and ablation catheter was at 3′O clock of the mitral annulus, quadripolar catheter at RV apex. [Quadripolar catheter placed at His region has displaced to CS proximal region during RFA]

**FIGURE 4:**
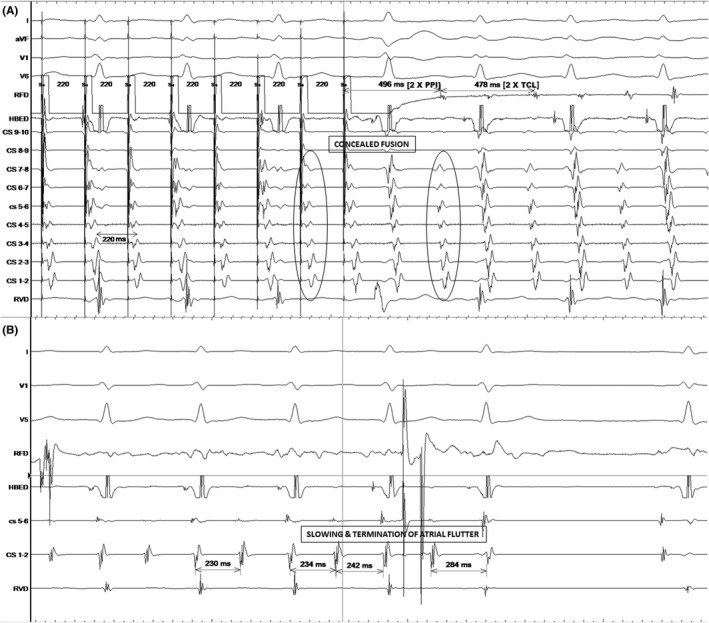
A, Represents surface ECG and intracardiac EGMs during entrainment from cavotricuspid isthmus (CTI). It shows the mechanism as cavotricuspid‐dependent atrial flutter. B, Represents surface ECG and intracardiac EGMs as radiofrequency energy was being delivered at the CTI. It showing slowing of the tachycardia followed by termination

